# Diversifying the research landscape: Assessing barriers to research for underrepresented populations in an online study of Parkinson’s disease

**DOI:** 10.1017/cts.2024.20

**Published:** 2024-02-01

**Authors:** Angie V. Sanchez, Juliana M. Ison, Helen Hemley, Jonathan D. Jackson

**Affiliations:** 1 Massachusetts General Hospital, Boston, MA, USA; 2 Harvard Medical School, Boston, MA, USA

**Keywords:** Research barriers, Parkinson’s disease, underrepresented groups, diverse representation, disparities, health equity

## Abstract

Despite federal regulations mandating the inclusion of underrepresented groups in research, recruiting diverse participants remains challenging. Identifying and implementing solutions to recruitment barriers in real time might increase the participation of underrepresented groups. Hence, the present study created a comprehensive dashboard of barriers to research participation. Barriers to participation were recorded in real time for prospective participants. Overall, 230 prospective participants expressed interest in the study but were unable to join due to one or more barriers. Awareness of the most common obstacles to research in real time will give researchers valuable data to meaningfully modify recruitment methods.

## Introduction

Medical studies with human volunteers are necessary to evaluate interventions focused on new medicines, products, and therapeutic procedures for healthcare and health behavior [1]. Despite the importance of clinical trials in advancing medicine, recruiting participants for these studies remains a challenge for researchers and physicians, especially among minority populations [2,3]. Even when federal regulations mandate the inclusion of underrepresented groups (e.g., racial and ethnic minority populations, rural populations, low-income populations, and populations with low educational attainment) in research [4,5], the involvement and systematic inclusion of these communities in clinical investigation remains disproportionately low [6,7]. Recruiting Underrepresented Groups (URGs) to research studies is crucial to more comprehensively understand diseases and treatment development as well as improve health care delivery [8]. Furthermore, engaging URGs in research is critical to significantly reducing health disparities and driving equity in health care delivery [9,10]. Regardless of the increasing awareness of barriers to research, investigators have continued to focus on the underrepresentation of minority populations, rather than specific or comparative strategies to overcome these barriers [11–14].

Community-based research has shown that collecting action communities, including social relations between local organizations and the surrounding community, helps build social cohesion, social trust, and participation – all fundamental to removing barriers to healthcare and research access [10,15,16]. Yet most previous research on diverse and inclusive research recruitment does not extend these findings to all URGs; nor does the extant literature develop strong, testable, and scalable engagement and recruitment frameworks for these individuals in these communities [17]. Critically and most surprisingly, no study has identified and implemented solutions to recruitment barriers in real time using the same prospective sample to our knowledge [17].

Hence, the Fostering Inclusivity in Research Engagement for Underrepresented Populations in Parkinson’s Disease (FIRE-UP PD) Study [18], a multi-site online study funded by the Michael J. Fox Foundation (MJFF), created a dashboard of barriers to research participation to comprehensively capture the multifaceted and multilayered barriers prohibiting prospective participants from participating in the study. This study was designed to mimic barriers to research participation in Fox Insight an online PD study with the Michael J. Fox Foundation to understand if research barriers can be manipulated through intervention to increase URG participation. The FIRE-UP PD Study was a stratified-randomized study in which eight sites were selected according to a proposed intervention. This study aimed to assess trust in and engagement toward PD research among URGs, increase participation of URGs to Fox Insight, and identify and disseminate methods and best practices to engage and recruit URGs in PD clinical research across eight months [18].

In this report, we outline barriers to recruitment in the FIRE-UP PD Study within the control and intervention groups. Through better understanding of the real-time barriers to clinical investigation in Parkinson’s disease, researchers can more strategically prioritize health equity and diversify the traditional research landscape through community-based approaches by eliminating these common barriers to research that routinely obstruct members of underrepresented groups from participating. Research barriers specific to the FIRE-UP PD Study that have been overcome will be described in our upcoming manuscript, which will detail all study findings.

## Methods

Methods for the FIRE-UP PD Study have been previously described in detail [18]. Briefly, the FIRE-UP PD Study asked eight participating sites to identify a URG or geographical region to measure changes in awareness and trust in Parkinson’s Disease (PD) research along with engagement and interest in PD protocols through the use of several surveys. The FIRE-UP PD Study measured changes in awareness and trust in PD research using the Trust in Medical Research Scale. Research engagement was measured through use of the Patient Engagement in Research Scale. Finally, interest in three hypothetical PD clinical trial protocols was measured, leveraging surveys developed by Dr. Allison Willis which were collectively titled the “PD Research Participation Survey” for the purpose of this study [18]. All participating sites were provided with tablets to perform surveys and collect data by using REDCap database a secure survey platform. Questionnaires for these surveys can be found in Sanchez *et al*., “Designing the Fostering Inclusivity in Research Engagement for Underrepresented Populations in Parkinson’s Disease study [18].” Surveys were collected for all sites at two separate time periods: pre-intervention and post-intervention, allowing the study team to evaluate changes in awareness after interventions. Interventions included developing educational tools to engage local communities, building partnerships within local PD communities, and recruiting stakeholders to reimagine medical and research information for the community. Additional information about interventions can also be found at Sanchez *et al*. [18].

The FIRE-UP PD Study also aimed to increase Fox Insight (an online study with MJFF) participation. Sites were randomly assigned to either the intervention or control condition. Interventions included developing educational tools to engage local communities, building partnerships within local PD communities, and recruiting stakeholders to reimagine medical and research information for the community [18]. Researchers adopted Picillo *et al*.’s framework [19] to systematically outline barriers to recruitment in a research barriers dashboard. Barriers fell within the following five major categories: infrastructure, nature of the research, recruiter characteristics, participant characteristics, and community with the first four categories developed by Picillo and colleagues [19], and the final category, community, being added by the current researchers to capture the team’s focus on community-centered recruitment approaches. The majority of the recorded barriers focused on infrastructure and participant characteristics as drivers of willingness to participate. See Table 1 for more information.

**Table 1.** List of tracked barriers as categorized by the modified Picillo et al.’s framework

Infrastructure	Nature of the research	Recruiter characteristics	Participant characteristics	Community
Lack of email	Other (descriptions included cognitive difficulties)	Language (only in English)	Low compensation	Lack of study partner
Lack of digital devices (laptop, cellphone, and tablet)				
		Mistrust	Lack of interest	
Lack of internet connection				
			Privacy/confidentiality concerns	
Lack of transportation				
Time commitment			No contact information	
Distance to study site				

Table 1 outlines the tracked study barriers, originally conceptualized by Picillo *et al*. and modified by the current authors through the addition of the “Community” category.

Prospective participants at all sites who were not recruited into the study had their barriers to participation recorded in real time via the research barriers dashboard developed for this project. The barriers dashboard did not require local Institutional Review Board approval as Personal Identifying Information was not collected. Instead, researchers only asked prospective participants for their reason to reject participation in this study. Barriers tracked included those related to language needs, digital limitations, trust, time commitment, transportation, contact information, as well as privacy concerns, and participants were able to describe other limitations not captured under the aforementioned categories. Research barriers dashboard was collected on a monthly basis by the Community Access and Research Engagement Research Center, located at the Massachusetts General Hospital, which served as the Recruitment and Engagement Coordinating Center for the FIRE-UP PD Study.

## Results

Multiple barriers to research participation were recorded for each prospective participant. A total of 488 participants were recruited to the FIRE-UP PD Study, with 295 participants recruited to intervention sites and 193 participants recruited to control sites. Two hundred and thirty prospective participants or the equivalent of 47% of all recruited participants expressed interest in the FIRE-UP PD Study but were otherwise unable to participate due to one or more of the tracked barriers. Demographic data could not be collected from prospective participants not able to participate as they were not enrolled in the study, but demographic data from recruited participants demonstrated that those able to participate were disproportionately White and highly educated with an annual household income of $50,000 or higher. Sanchez *et al*. [18] describe the demographic composition of enrolled participants for the FIRE-UP PD Study in more detail.

**Figure 1.**
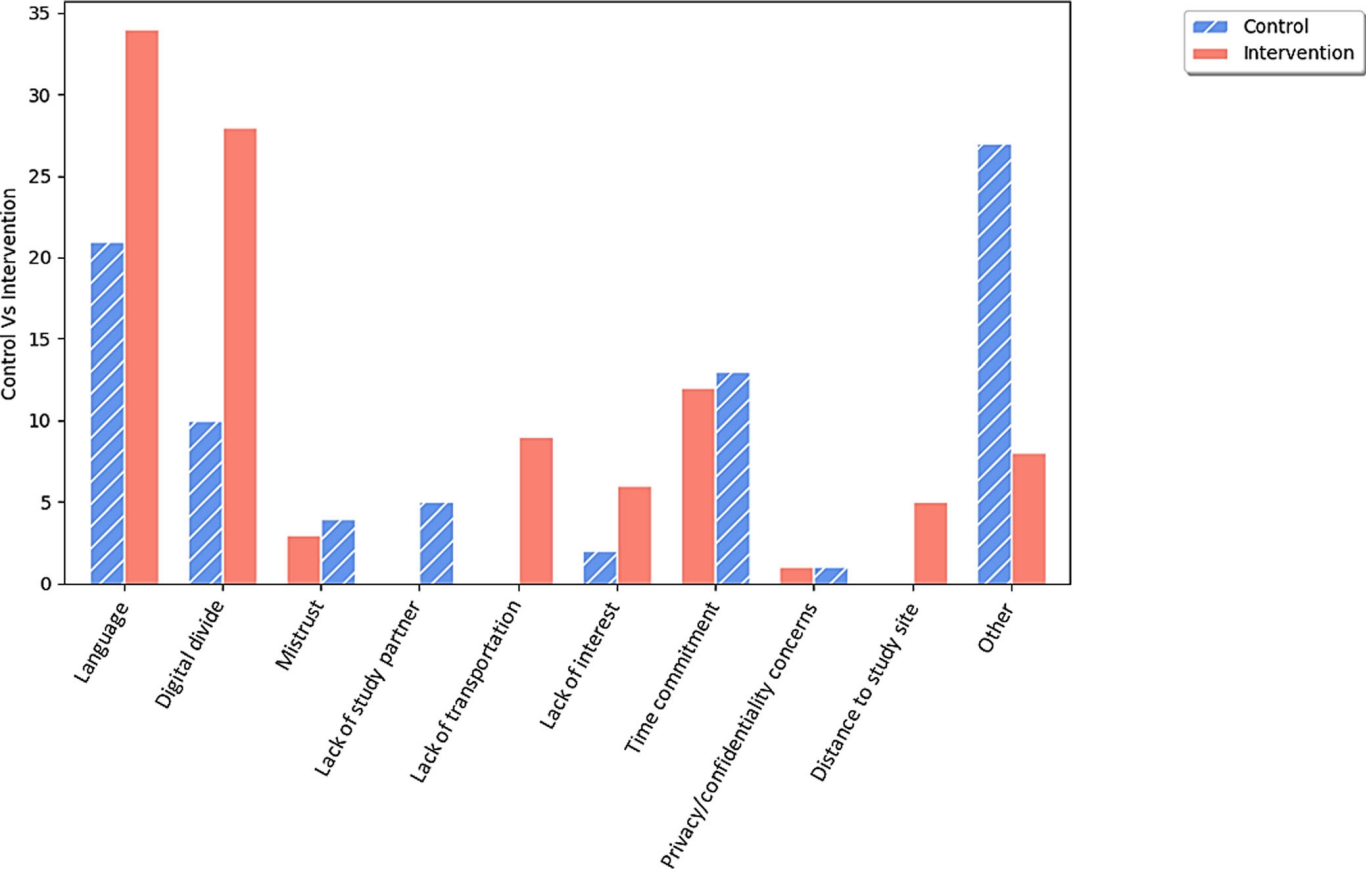
Barriers to recruitment separated by control and intervention site conditions. Depicts the captured barriers to study recruitment, differentiated by control and intervention site conditions.

**Figure 2.**
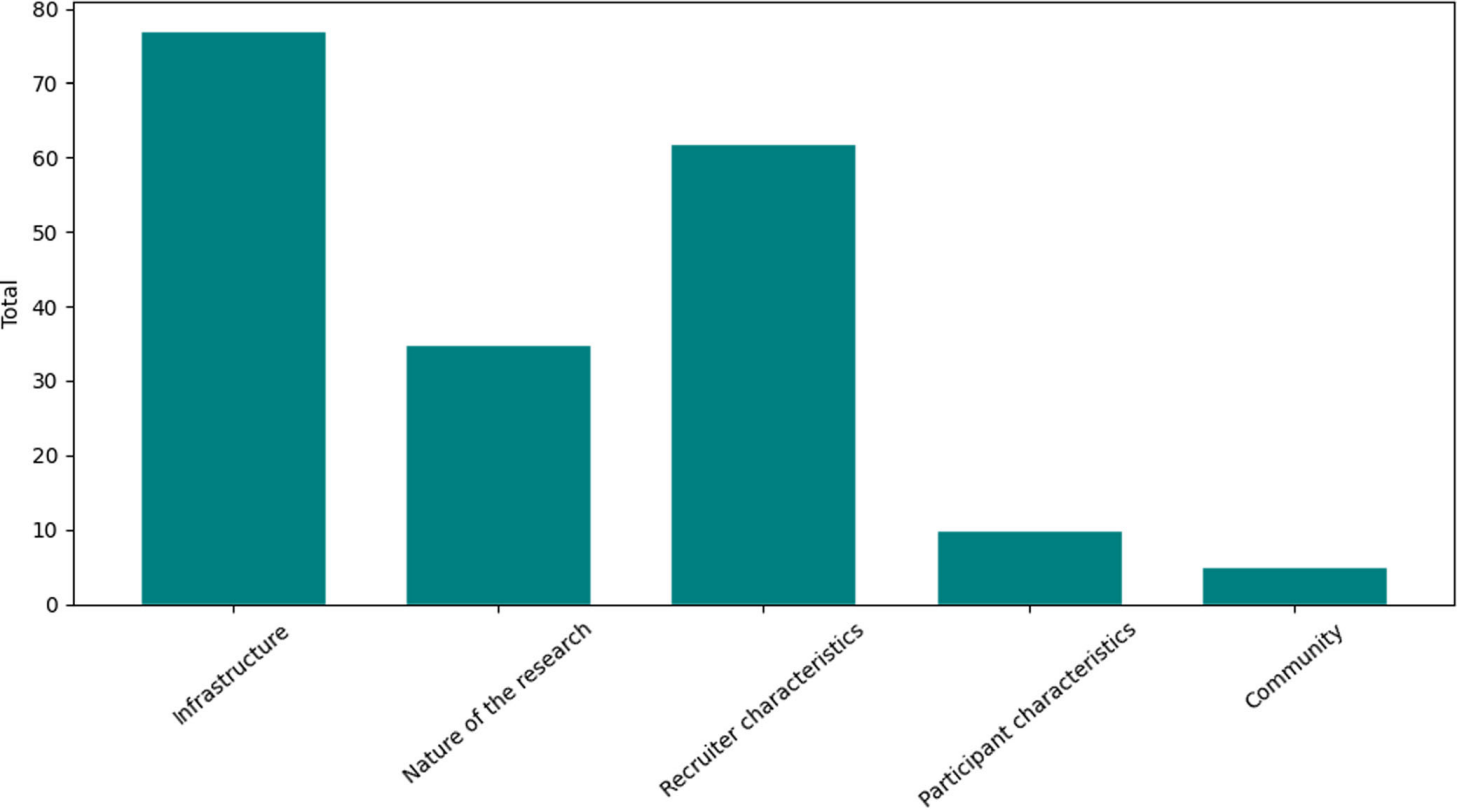
Barriers to Fostering Inclusivity in Research Engagement for Underrepresented Populations in Parkinson’s Disease categorized by Picillo *et al*.’s framework. Summarizes and categorizes the tracked barriers across the study into the modified Picillo *et al*.’s framework for research barriers.

See Figure 1 for more information on barriers grouped by control vs. intervention condition and Figure 2 for barriers categorized by Picillo *et al*.’s aforementioned framework [19].

Language barriers were the most frequently reported barriers across both intervention and control sites accounting for 29% of all recorded study barriers, given the study was only conducted in English to recreate the same barriers held by Fox Insight for consistency. Digital access barriers, i.e., lack of email, lack of digital device(s), and/or lack of internet connection, were the next most experienced across all sites comprising 20% of all barriers, while barriers related to cognitive and physical health (captured in the “Other” category descriptions) were the third most cited barrier across sites, accounting for about 19% of all reported barriers. Interestingly, mistrust, a frequently evoked research barrier [19], was acknowledged with low frequency (4% of all study barriers) in the FIRE-UP PD Study. Both low compensation and no contact information were not indicated by any prospective participants as barriers to research. Based on Picillo *et al*.’s study barriers framework, infrastructure barriers comprised the highest percentage of barriers to research participation (41%) while barriers related to recruiter characteristics were the next highest percentage of barriers (33%), and barriers related to the nature of the research constituted the third highest category of barriers (19%). Barriers related to participant characteristics and the community comprised the final 8% of barriers captured by prospective participants otherwise unable to participate. See Figure 2 for more information.

## Discussion

Results revealed barriers categorized as relating to infrastructure and recruiter characteristics in Picillo *et al*.’s framework to be the most commonly cited [19]. This is due to the profound quantity of prospective participants who cited digital access and language barriers, which fell within these two categories. Further investigation may more deeply explore the relationship between infrastructure and recruiter characteristics to individual participant barriers to mitigate these structural and interpersonal obstacles.

Limitations include the breadth and scope of barriers collected. Researchers chose to focus on 13 frequently cited barriers based on the literature, with an additional option for “Other” barriers. Thus, the breadth of barriers captured was limited by these criteria. Additionally, as this project specifically focused on PD, generalizability may be somewhat limited within this domain. Thus, the authors encourage more research to be done to replicate and corroborate these results in other sectors.

Capturing research barriers to prospective participants in real time is relatively novel. Through this practice as well as the accompanying systematization of the data into a modified version of the Picillo *et al*. framework [19] for interpretation, researchers were able to better understand pressing barriers to online Parkinson’s disease research. After analyzing results from the entire study, comprehensive results – including barriers overcome by those who did participate in the FIRE-UP PD Study – will be reported for future possible replication.

Many researchers continue to look for simple solutions to recruit URGs to research studies despite several interconnected barriers reported in the literature [11–13,16,18,20] which disproportionately affect URG research participation. Consequently, by continuing to focus on recruitment as an outcome over patient-centered measures of engagement, trust, and/or empowerment, the notable lack of representation among URGs in clinical trials will likely persist. By enhancing comprehension and interpretation of these interconnected obstacles hindering prospective participants from becoming involved in research, investigators can be better equipped to modify research recruitment methods as needed to develop a more localized approach to reach the populations that they hope to recruit, as there is no single activity that will allow for sustained or easy trial recruitment overall. Subsequently, by addressing and eliminating frequent barriers to clinical investigation currently being experienced, researchers may better promote and prioritize health equity in traditional research spaces by expanding studies to participants historically excluded through common research barriers. Through this intentional expansion of research to URGs through community-based methods, harmful patterns of exclusionary research may begin to be broken and recalibrated to address the needs of the community at large.
